# Resting metabolic rate is repeatable, but does not affect call characteristics, in the gray treefrog *Hyla chrysoscelis*

**DOI:** 10.1242/jeb.250570

**Published:** 2025-07-23

**Authors:** Phoebe Will, Elena Lawson, Zashri Cocheran, Michael S. Reichert

**Affiliations:** ^1^ Department of Integrative Biology, Oklahoma State University, Stillwater, OK 74078, USA; ^2^ Department of Biology, Stephen F. Austin State University, Nacogdoches, TX 75965, USA

**Keywords:** Acoustic communication, Metabolic rate, Anuran, Behavioral energetics, Sexual selection, Repeatability

## Abstract

Consistent among-individual variation in behavior is widespread and often has consequences for fitness. However, the mechanistic basis of repeatable variation in behavior is less understood. Metabolic rate is a likely candidate to drive repeatability in behavior because energy metabolism can limit behavioral expression. There are competing hypotheses for the relationship (or lack thereof) between levels of maintenance metabolism such as resting metabolic rate (RMR) and the expression of behaviors. On the one hand, RMR may show a negative relationship with behavior if higher RMR limits energy that can be allocated to other processes. On the other hand, RMR may positively correlate with behavior if high RMR leads to greater energy production ability. To test these hypotheses, we examined the relationship between RMR and repeatable, highly energetically costly sexual signals in male Cope's grey treefrogs (*Hyla chrysoscelis*). We recorded individual male acoustic advertisement calls in the field and measured their RMR the following day. We made repeated measurements of RMR across multiple captures of the same individuals to assess the repeatability of metabolic rates, and whether consistency in RMR decreases over time. There was no evidence that RMR affected call characteristics in *H. chrysoscelis*. Nevertheless, RMR was significantly repeatable. We found that RMR decreased across the breeding season, which reduced the consistency in RMR measurements of the same individual over time. We conclude that calling in *H. chrysoscelis* does not provide information to mates or rivals on male RMR, although other aspects of metabolism may still drive individual variation in calling.

## Teaser

**Summary:** Resting metabolic rate does not explain among-individual variation in calling in gray treefrogs, but metabolic rate was repeatable.

## INTRODUCTION

Consistent differences among individuals in behavior are common, and have important consequences for fitness (Moiron et al., 2020). Even for plastic traits, individuals often show repeatable behavioral variation, which can lead to differences in foraging success, predator avoidance and mate attraction (Dall et al., 2012; Réale et al., 2007; Wolf and Weissing, 2010). Although the consequences of repeatable behavioral variation are well documented, there is still an open question of what the primary mechanistic drivers of this variation are (Laskowski et al., 2022). Even at this level, much of the focus has been on whether heritable genetic variation or long-term environmental effects generate individual variation in behavioral phenotypes (Dochtermann et al., 2019; van Oers and Mueller, 2010). The immediate mechanistic drivers of consistent among-individual differences in behavior are less well understood, but are an important target of investigation (Biro and Stamps, 2010; Carere et al., 2010). Identifying the mechanism behind behavioral variation could reveal the types of genes or environmentally sensitive traits that are involved, and potentially link these traits to performance and fitness (Pettersen et al., 2016). Furthermore, for sexually selected signaling behaviors, identifying the mechanism that generates variation in signals could indicate the true target of selection. In indicator models of mate choice, certain signal traits are preferred because they are correlated with physiological processes that contribute to individual quality and that could be inherited by offspring (Andersson, 1994; Lailvaux and Irschick, 2006). Although there have been many proposals that physiology drives repeatable behavioral variation, there is still much debate about which types of traits are likely to be associated, and what is the expected direction and magnitude of these associations.

List of abbreviations

BP: barometric pressure
*F*e_O_2__: excurrent fractional O_2_ concentration
FR: flow rate
FR_d_: flow rate corrected for water vapor
RMR: resting metabolic rate
WVP: water vapor pressure

One physiological mechanism that is especially likely to explain variation in behavior is metabolic rate (Holtmann et al., 2017; Niemelä and Dingemanse, 2018). Energy from metabolism is required for all behaviors, and metabolic rate determines the energy available to be allocated to behavioral expression (Biro and Stamps, 2010). Metabolic rates themselves often show consistent variation among individuals (Nespolo and Franco, 2007; White et al., 2013). Even after accounting for traits that strongly affect metabolic rate, such as body mass, age, sex, temperature and reproductive status (McNab, 2008), conspecifics can have several-fold differences in metabolic rate (Burton et al., 2011; Konarzewski and Książek, 2013). However, metabolism is a complex trait that can be quantified at multiple levels from the activity of individual mitochondria to whole-organism levels of gas exchange under different conditions (Pettersen et al., 2018). Much work has focused on maintenance metabolic rates, which characterize an individual's energy use for basic survival functions, and how they are correlated with life history traits and behavior (Larivée et al., 2010; Ricklefs et al., 1996; Speakman et al., 2004; Steyermark, 2002). For example, crickets with higher standard metabolic rates were slower to exit a shelter, suggesting a trade-off between maintenance and allocation of energy to behavior (Careau et al., 2018).

Although there is now substantial evidence that maintenance metabolism affects behavior, there is still debate about the expected direction of such effects, and more broadly under what circumstances or for what types of behaviors these effects are likely to occur (Burton et al., 2011; Careau and Garland, 2015; Mathot and Dingemanse, 2015; Mathot et al., 2019). This debate is echoed by the broader question of whether and how metabolic rates affect fitness (Arnold et al., 2021). The competing hypotheses have been described as energy management models, and ultimately differ in whether maintenance metabolic rate limits or enhances the scope for behavioral expression (Biro et al., 2018). The allocation model proposes a negative relationship between maintenance metabolic rate and the amount of energy invested into behavior, because a higher energetic requirement for maintenance would trade off against energy that could be allocated to other traits (Biro et al., 2020). In storm petrels, the allocation model was supported for males, although not females. Eggs hatched sooner and the resulting chicks grew faster when the male parent had a lower basal metabolic rate – suggesting that males with low maintenance energetic costs could devote more energy to caring for their offspring (Blackmer et al., 2005). In contrast, the performance model proposes a positive relationship between maintenance metabolic rate and behavior (Biro and Stamps, 2010; Nilsson, 2002). This model is based on the idea that individuals with higher maintenance metabolic rate have a larger metabolic engine and therefore have more energy to allocate to behaviors (Bennett and Ruben, 1979; Taigen, 1983). For example, bank voles had higher reproductive success (total number of offspring) when they had a higher basal metabolic rate because they were able to invest more energy into parental care (Boratyński and Koteja, 2010). Finally, the independent model proposes no relationship between maintenance metabolic rate and behavior; for instance, in brook charr, no relationship was found between time spent foraging and resting metabolic rate (Farwell and McLaughlin, 2009). As there is support for all three models in different species, it is likely that the relationship between metabolic rate and behavior is species, context or behavior dependent. Thus, it is important to expand the range of taxa and behaviors in which metabolism–behavior relationships are investigated.

The hypothesis that consistent among-individual differences in metabolic rate lead to consistent among-individual differences in behavior assumes that there are consistent among-individual differences in metabolic rate (Biro and Stamps, 2010). This variation can be quantified using the repeatability coefficient (Nakagawa and Schielzeth, 2010), where high values of this coefficient indicate a substantial among-individual component to the trait variance (Laskowski et al., 2022). Although maintenance metabolic rate is generally repeatable, many taxa are understudied, and repeated metabolic rate measurements are often taken only over very short time intervals (Nespolo and Franco, 2007). The repeatability of traits, including metabolic rates specifically, often decreases when there is more time between measurements and tends to be lower in free-living individuals (Auer et al., 2016; White et al., 2013). Therefore, more studies are needed of metabolic rate repeatability in underrepresented taxa, and in which the consistency of metabolic rate is demonstrated over longer time periods and from wild populations.

Sexual signaling is likely to be related to metabolic rate. Sexual signaling behaviors often show consistent among-individual variation and are also often extremely energetically costly (Kotiaho et al., 1998; Reinhold et al., 1998; Ryan, 1988; Welch et al., 2014). These relationships suggest that individual variation in maintenance metabolic rate may drive variation in sexual signaling if it affects the energy available for signal development or behavioral expression. However, much of the evidence for the covariance between sexual signals and maintenance metabolism is indirect and based on allometric scaling or comparisons between sexes or populations varying in sexual dimorphism (Curlis et al., 2021; Kotiaho, 2001; Somjee, 2021; Somjee et al., 2022). Advertisement calling by male anurans is a prototypical energetically costly sexually selected display (Ryan, 1988; Sullivan and Kwiatkowski, 2007; Wells, 2001). In the gray treefrog, *Hyla versicolor*, for instance, calling is energetically expensive, and increases in call duration or call rate, both traits under strong directional selection by female mate choice, incur an energetic cost that may push males to the limits of their performance capabilities (Reichert and Gerhardt, 2012; Taigen and Wells, 1985; Wells and Taigen, 1986). The closely related species *Hyla chrysoscelis* has not been investigated for relationships between metabolism and calling, but such a relationship is likely because females prefer energetically costly call characteristics (Tanner et al., 2017; Ward et al., 2013), and males show repeatable variation in these same characteristics (Reichert et al., 2024). However, anurans generally have low maintenance metabolic rates, and metabolic rate increases several-fold during calling (Wells, 2007), raising questions about whether maintenance metabolism would be expected to affect the expression of calling behavior.

We measured individual variation in calling behavior and resting metabolic rate (RMR) in Cope's gray treefrog, *H. chrysoscelis*, and tested four hypotheses. First, we measured RMR and calling in the same individuals to test which energy management model best explains the relationship between RMR and calling in *H. chrysoscelis*. Second, we tested what biological and environmental factors affect RMR. Third, we tested whether there are repeatable among-individual differences in RMR in *H. chrysoscelis*. If RMR is repeatable, this could be a mechanism contributing to repeatable variation in calling and potentially indicate that RMR is a target of sexual selection in this species. Fourth, we tested whether the consistency of RMR measurements decreases with an increasing number of days between the initial and final measurement of RMR.

## MATERIALS AND METHODS

All procedures were carried out with the approval of the Institutional Animal Care and Use Committee at Oklahoma State University (protocol number AS-22-22) and with permits from the Oklahoma Department of Wildlife Conservation. The health of all frogs was monitored throughout the experiment, and we observed no mortality or adverse effects in this study.

### Acoustic recordings

We collected male *Hyla chrysoscelis* Cope 1880 from seven ponds in Payne County, OK, USA, during their breeding season, from May through July in 2023 and 2024. Individual male *H. chrysoscelis* producing advertisement calls in the field were located between 20:00 and 24:00 h. Once located, we recorded 30 calls per frog using a Zoom H6 digital recorder and a Sennheiser ME66 microphone. Recordings were formatted as 16-bit .wav files, with a sampling rate of 44.1 kHz. Because *H. chrysoscelis* are ectothermic, many of their call characteristics are affected by temperature (Gayou, 1984; Prestwich, 1994), so we measured their skin-surface temperature using a Fluke 62 MAX+ IR thermometer immediately after the recording and included temperature as a factor in all analyses with call characteristics as the dependent variable to correct for its effects (see ‘Statistical analysis’). We measured the temperature by aiming the thermometer at the frog and holding it as close as possible to the frog's body, always less than 1 m away and usually much closer. Skin-surface temperatures ranged from 14.8 to 29.8°C (mean±s.d.=21.7±2.7°C). Individuals were then caught by hand and placed in a small, ventilated container with a small amount of pond water and transported back to the lab. We measured their RMR in the lab the day after capture (see below). Afterwards, and on the same day as the RMR measurement, we measured body mass and tagged each individual with a unique visible implant elastomer code for individual identification. Tags were injected underneath the skin on the ventral surface of the feet (6 total, up to 2 tag colors per foot). Frogs were unanesthetized and were restrained by hand during this procedure. Frogs were then rereleased into their pond of origin that same night.

We used Raven Pro version 1.6 software (Cornell Laboratory of Ornithology) to analyze male call characteristics. Specifically, for each call we measured call duration (time from beginning to end of the call), pulse number (the number of pulses per call) and dominant frequency. We also measured call rate on a call-by-call basis as the reciprocal of the amount of time between the onset of a call and the onset of the next call (final call of the recording excluded). We calculated call effort as the call duration multiplied by the call rate, again on a call-by-call basis. For the 2023 data, 92 recordings were analyzed independently by two different researchers (E.L. and Z.C.) so that we could estimate inter-observer reliability of each measurement. The 2024 data were analyzed by a single researcher (P.W.).

### RMR measurement

Metabolic rate measurements were collected during the day (between 09:00 and 14:00 h) to ensure individuals were at rest and not producing advertisement calls. Each individual was placed in one of eight small sealed 200 ml glass Mason jar chambers within a Percival environmental chamber. One chamber was left empty each trial to serve as a control chamber. Individuals were acclimated in the chambers for 15 min before recording began. We measured metabolic rates at an air temperature of 25°C, which is similar to the average nightly breeding season temperature in our population. The lights in the environmental chamber were left on because measurements were made during the day and this species is more active at night. Because individuals were measured during the breeding season and we could not ensure that they were post-absorptive, we classify our measurement of maintenance metabolism as RMR, as opposed to standard metabolic rate, which requires that measurements be taken from post-absorptive non-reproductive animals.

We used a push mode respirometry system (Lighton, 2019). First, Bev-a-line tubing passed air from the building ventilation system through two sealed empty buckets (19 and then 13.2 liters) to buffer fluctuations in oxygen levels (Lighton and Halsey, 2011). This air was then pulled into a Sable Systems PP-2 Dual Channel Pump. From the pump, air was pushed through a column containing soda lime to remove CO_2_ and Drierite to remove water vapor, and then through a second tube of Ascarite to remove any remaining CO_2_. All chemical scrubber inlet and outlet tubes were filtered using cotton. To prevent frogs from dehydrating, the air then was rehydrated by pushing it through Nafion tubing in a bottle of distilled water; note that we did not measure the humidity in the chamber itself. Afterwards, we used a manifold to create a separate air stream on each of the eight input channels on a Sable Systems Flowbar-8 Mass Flow Meter to control the flow rate of air going into each chamber. Flow rate was adjusted to approximately 100 ml min^−1^ during trials. Tubes connected to the out channel of the flow meter then pushed the air into each individual respirometry chamber; tubes were sealed with glue to prevent mixing with outside air. The air input into the chamber was directed towards a baffle to improve mixing. Air then exited the chambers through a second tube and entered a Sable Systems RM-8 Flow Multiplexer. The multiplexer was set to automatically switch between channels every 15 min, and after all channels had been recorded, we repeated the entire cycle two more times such that each channel was recorded three times, and the recording always finished with another 15 min from the baseline chamber. We will refer to each 15 min recording period as a repetition and the set of three repetitions for an individual on one day of measurement as a trial. Note that the duration of the entire recording and the time between measurements for each individual depended on the number of frogs being measured. The air from the multiplexer was then passed through a Sable Systems Field Metabolic System. We analyzed water vapor pressure first, then passed the output through a tube of magnesium perchlorate (Anhydrone) to remove H_2_O before measuring CO_2_. The output from the CO_2_ analyzer then passed through separate tubes of Ascarite and Anhydrone to remove CO_2_ and residual water vapor prior to measuring O_2_. Measurements of O_2_, CO_2_, barometric pressure (BP), water vapor pressure (WVP) and flow rate were recorded in ExpeData software (Sable Systems International). The respirometer was calibrated weekly using pure nitrogen gas and a gas mixture of CO_2_ in nitrogen gas (0.0959% CO_2_ with 2% analytical uncertainty), and left on continuously during the field season to avoid long warm-up times and reduce drift on the oxygen sensor.

### RMR analysis

We calculated RMR in ExpeData software (Sable Systems). Prior to measuring rates of oxygen consumption (*V̇*_O_2__) and CO_2_ production (*V̇*_CO_2__), raw data were transformed using a custom ExpeData macro. We corrected for lag between the three gas analysis channels using a combination of automatic correction by the ExpeData Lag Correct function, and manual correction in cases where the function did not clearly adjust the lag. We used uniform tubing lengths for all chambers to reduce variation in lag across chambers. We used the baseline correction function in ExpeData to correct for drift in the O_2_ and CO_2_ channels, resulting in a flat baseline. We calculated proportional O_2_ consumption by flipping the O_2_ data in sign and dividing by 100, and proportional CO_2_ production was also calculated by dividing by 100. Finally, we corrected the flow rate measurement to account for the water vapor that was present in the system after rehydrating the air (Lighton, 2019) using the equation:
(1)

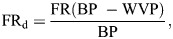
where FR_d_ is the corrected flow rate, FR is the measured flow rate prior to correction, BP is the barometric pressure in kPa and WVP is the water vapor pressure in kPa. We then calculated metabolic rate as the rate of oxygen consumption, *V̇*_O_2__, in units of ml h^−1^ as:
(2)

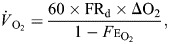
where ΔO_2_ is the difference between the incurrent and excurrent fractional O_2_ concentrations and *F*e_O_2__ is the excurrent fractional O_2_ concentration (Withers, 2001).

All recordings were visually inspected for smoothness. Repetitions where individuals appeared to be active, as inferred by sudden changes in *V̇*_O_2__, were discarded. To calculate an individual's RMR, we used ExpeData to search the useable repetitions for areas that had relatively stable *V*_O_2__ values, and within those areas searched for a region with the lowest *V̇*_O_2_ _values (indicating minimal metabolic rate). Specifically, we used the 450 s interval of the repetition with the lowest standard deviation in *V̇*_O_2__, and within that interval, we found the 60 s interval with the lowest mean *V̇*_O_2__. The individual's RMR was defined as the lowest of these mean *V̇*_O_2__ values across the three repetitions.

### Statistical analysis

We measured RMR from a total of 100 individual *H. chrysoscelis*, with 143 unique trials because some individuals were captured and measured multiple times. We removed RMR data for repetitions, or in some cases whole trials, that had technical issues or because individuals appeared to be active during recording (90 total repetitions removed). After filtering, we had data on RMR from 136 unique trials for 99 individuals (21 individuals measured twice and eight individuals measured three times). Some call recordings were not useable and there was one call recording with no temperature measurement, so our sample for any analyses including call characteristics was 122 unique recordings of 95 individuals (19 individuals recorded twice and four individuals recorded three times). Statistical significance was evaluated using an alpha of 0.05. Raw data and the code used to analyze the data and generate figures are available at https://doi.org/10.6084/m9.figshare.28525880.

#### Effects of RMR on call characteristics

We tested for relationships between RMR and call characteristics using separate linear mixed models with each of the five call characteristics (call duration, pulse number, dominant frequency, call rate and call effort; in all cases as the mean value from the recording) as the dependent variable and fixed factors for RMR, body mass (g), temperature while calling, calendar day and year (categorical, 2023 or 2024). Individual identity was included as a random effect. We calculated models using the lme4 package version 1.1-35.5 (Bates et al., 2015) in R version 4.4.1 software (https://www.r-project.org/).

We calculated inter-observer reliability of the call characteristic measurements from 92 recordings from 2023 that were analyzed independently by two different individuals (*N*=2758 calls). We calculated the correlation coefficient between the two measurements for each individual call, along with the slope of the linear regression to assess whether it was close to the ideal value of 1.

One of our aims in this study was to determine whether the consistent among-individual variation in calling observed in this population is explained by consistent among-individual variation in RMR. We based this aim on a previous demonstration of repeatability of multiple call characteristics in a large sample of males from our study population (Reichert et al., 2024). We confirmed that the males in the present study also showed repeatability of call characteristics by calculating repeatability coefficients for the mean values from each recording of each of the five call characteristics using the rptR version 0.9.22 package in R (Stoffel et al., 2017), with temperature as a covariate and individual identity as a random effect. These results are reported in Table S1. Finally, we note that the RMR values used as a factor in this analysis were not corrected for body size. Because metabolic rate covaries with body mass, it is common for studies of metabolic rate variation to calculate a mass-adjusted metabolic rate. However, in our study, the aim was to test whether gross energy expenditure affects calling, and the energy management models that we tested are based on relationships between energy required for maintenance versus energy that can be allocated to behavior. Correcting for body mass could obscure these relationships. Therefore, our approach follows previous work on effects of energy expenditure on behavior and life history in using absolute RMR as the variable of interest (Mathot et al., 2015; Tieleman et al., 2009). Furthermore, with the exception of dominant frequency, call traits in *H. chrysoscelis* do not scale with body mass and so the mass-scaling of RMR was not a confounding factor in these analyses (Ward et al., 2013).

#### Factors affecting RMR

We tested effects on RMR using a linear mixed model with RMR as the dependent variable, individual identity as a random effect, fixed effects of calendar day, year and body mass, and a quadratic effect of calendar day. The quadratic term was not significant and we did not retain it in the final model. We ran an additional model with log_10_-transforms for both RMR and body mass because mass often scales non-linearly with RMR (Clarke et al., 2010).

#### Repeatability of RMR

We estimated the repeatability of RMR by calculating the repeatability coefficient using rptR, with mass (g) and calendar day as covariates and individual identity as a random effect. RMR was treated as a Gaussian variable.

#### Effects of time between trials on consistency of RMR

To determine how consistent RMR is within individuals depending on the amount of time between their first and last measurement, we used a linear regression with the number of days between the first and the final trial as the explanatory variable and the absolute value of the difference between the RMR measured on the first trial and the RMR measured on the last trial as the dependent variable. We used the absolute value of the difference between first and last RMR because we were interested in the magnitude of change in RMR with increased time between trials, not necessarily whether that change was positive or negative. Note that we used first and last measurements (as opposed to second, for individuals captured three times) to maximize the difference in the amount of time between repeat measures. However, we analyzed within-year variation only, and therefore excluded three repeated measures that were taken across the two years. These three points were strong outliers and we did not have enough data to properly examine cross-year effects. If individuals had three captures across years, the two data points within a year were kept while the one data point in the other year was excluded. Because we found that RMR varied strongly with day of the year (see Results), we ran a second version of this analysis in which we calculated the residual RMR from a linear regression of RMR on calendar day, and then used the absolute value of the difference between these residual values on the first and last trials as the dependent variable. Thus, this analysis tests whether time between measurements still affects the consistency of RMR within individuals after controlling for time of year effects.

## RESULTS

The mean RMR across the 136 trials for male *H. chrysoscelis* was 0.92 ml O_2_ h^−1^ (s.d.=0.38), and 0.18 ml O_2_ h^−1^ g^−1^ (s.d.=0.06) when mass corrected by dividing by body mass. There was high inter-observer reliability for all five call characteristics (Table S2). Therefore, for recordings that were analyzed by both observers, we used the results from one observer (E.L.), but confirmed that using results from the other observer (Z.C.) did not have qualitative effects on our findings.

### Effects of RMR on call characteristics

**Fig. 1.**
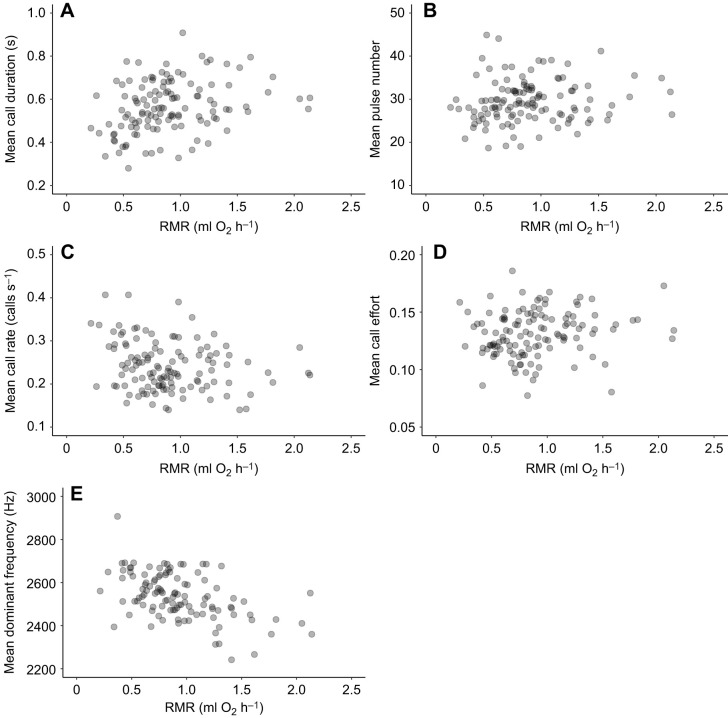
**Plot of the relationships between resting metabolic rate (RMR) and call characteristics in *Hyla chrysoscelis*.** (A) Call duration, (B) pulse number, (C) call rate, (D) call effort and (E) dominant frequency. Each point represents data from one measurement of an individual. Note that some individuals contributed multiple data points because they were captured and measured more than once.

There was no significant relationship between RMR and any of the call characteristics of male *H. chrysoscelis* (all *P*>0.05; Fig. 1, Table 1). However, multiple other factors included as covariates had significant effects on some call characteristics. Pulse number was significantly influenced by calendar day and year (*P*=0.03), call period and call duration were affected by temperature while calling (*P*<0.005) and year (*P*<0.02), and dominant frequency was affected by calendar day and mass (*P*<0.001) (Table 1).

**Table 1.** Linear mixed model statistics for the effects on call characteristics

Call characteristic	Factor	Estimate	s.e.m.	*t*	*P*
Call duration (s)	RMR (ml O_2_ h^−1^)	0.02	0.03	0.79	0.43
	Temperature (°C)	−0.03	0.004	−6.46	<0.001*
	Calendar day	−0.001	0.0009	−1.73	0.09
	Mass (g)	−0.009	0.01	−0.90	0.37
	Year (2024)	0.07	0.02	3.26	0.002*
Pulse number	RMR (ml O_2_ h^−1^)	0.72	1.46	0.49	0.62
	Temperature (°C)	−0.07	0.21	−0.35	0.73
	Calendar day	−0.10	0.05	−2.21	0.03*
	Mass (g)	−0.49	0.56	−0.88	0.38
	Year (2024)	2.56	1.18	2.17	0.03*
Call rate (calls s^−1^)	RMR (ml O_2_ h^−1^)	−0.012	0.015	−0.81	0.42
	Temperature (°C)	−0.010	0.002	4.51	<0.001*
	Calendar day	0.0001	0.0005	0.27	0.79
	Mass (g)	0.006	0.006	1.01	0.32
	Year (2024)	−0.03	0.01	−2.46	0.02*
Call effort	RMR (ml O_2_ h^−1^)	−0.00117	0.0061	−0.19	0.85
	Temperature (°C)	−0.0009	0.0009	−1.06	0.29
	Calendar day	−0.0003	0.0002	−1.61	0.11
	Mass (g)	0.0009	0.0023	0.40	0.69
	Year (2024)	−0.0008	0.0049	−0.17	0.87
Dominant frequency (Hz)	RMR (ml O_2_ h^−1^)	−26.80	22.93	−1.17	0.25
	Temperature (°C)	1.63	3.44	0.47	0.64
	Calendar day	2.39	0.71	3.37	0.001*
	Mass (g)	−63.96	8.98	−7.12	<0.001*
	Year (2024)	10.32	17.97	0.57	0.57

Year was entered as a categorical variable, with 2023 as the reference value. Individual identity was a random effect. The estimate of the effect, its s.e.m., the value of the test statistic (*t*) and the *P*-value are from the linear mixed model output. Statistically significant *P*-values are denoted with an asterisk.

### Factors affecting RMR

**Fig. 2.**
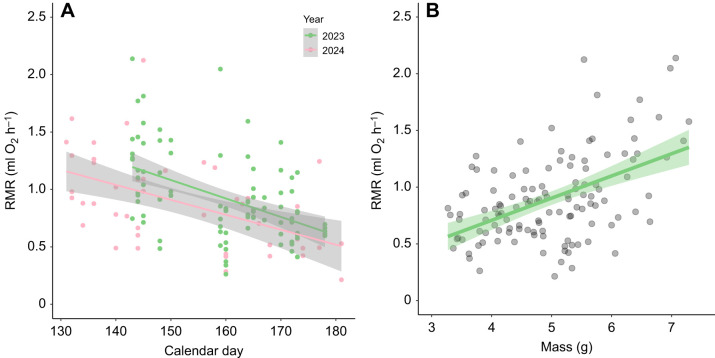
**The effects of calendar day and year and body mass on RMR in male *H. chrysoscelis*.** (A) Calendar day and year. (B) Body mass. The 2023 season is shown in green and the 2024 season in pink. In A, each point represents an individual's RMR on a given day and each trend line shows a negative, linear relationship for that year. The shaded areas around each line represent the 95% confidence interval. Note that some individuals contributed multiple data points because they were captured and measured more than once, so the regression lines are for illustration purposes only.

RMR significantly decreased over the breeding season, as indicated by a significant negative effect of calendar day, and RMRs were lower in 2024 than in 2023 (Table 2, Fig. 2A). There was no quadratic effect of calendar day on RMR (*P*_linear_<0.001, *P*_quad_=0.47). RMR increased with mass (Table 2, Fig. 2B), and there was no obvious improvement in model fit when RMR and body mass were log_10_-transformed (Fig. S1).

**Table 2.** Linear mixed model statistics for factors affecting RMR

Factor	Estimate	s.e.	*t*	*P*
Calendar day	−0.01	0.0021	−5.10	<0.001*
Mass (g)	0.18	0.03	5.94	<0.001*
Year (2024)	−0.24	0.06	−3.70	<0.001*

Statistically significant *P*-values are denoted with an asterisk.

### Repeatability of RMR

**Fig. 3.**
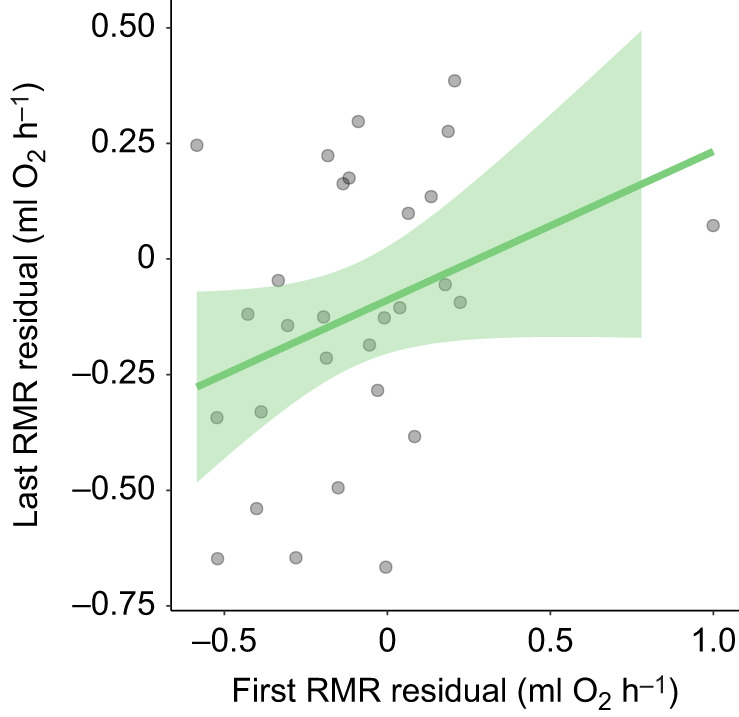
**Illustration of consistent among-individual differences in RMR as a scatterplot.** Each point represents an individual and corresponds to residuals of the first and last measurement of its RMR after accounting for the fixed effects of mass and calendar day from a mixed model with individual identity as a random effect. Trend line is from a linear least squares regression to illustrate the pattern; the shaded area around the line represents the 95% confidence interval. *N*=29 individuals. A scatterplot showing raw RMR values that were not adjusted for fixed effects is shown in Fig. S2.

There were consistent among-individual differences in RMR (Fig. 3). RMR was significantly repeatable, with a repeatability coefficient of 0.4 (95% CI [0.109, 0.65], *P*=0.008).

### Effects of time between trials on consistency of RMR

**Fig. 4.**
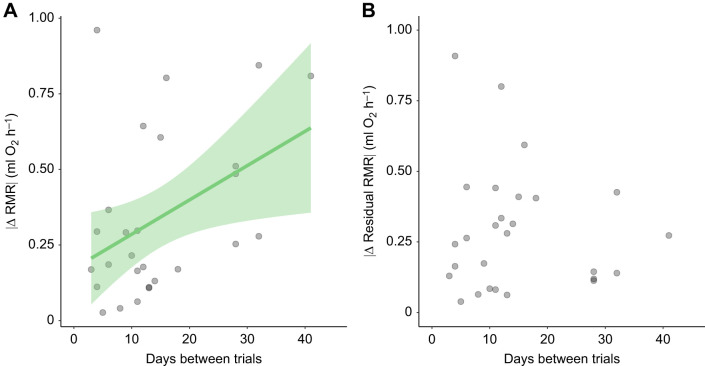
**The effects of time between measurements on consistency in resting metabolic rate.** (A) Relationship between the absolute value of the difference in RMR between the first and last trials, and the number of days between the first and last trials. (B) Relationship between the absolute value of the difference in the residuals from a regression of calendar day on RMR between the first and last trials and the number of days between the first and last trials. Accounting for calendar day removes the effect of days between trials on the change in RMR. Line in A is from a linear least squares regression and shaded area is the 95% confidence interval. Line not shown in B because the relationship was not statistically significant.

The absolute value of the difference between RMR measurements increased significantly as the number of days between the first and last measurements increased (*N*=27, β=0.011, *t*=2.36, *P*=0.027) (Fig. 4A). However, when we analyzed this relationship using the absolute value of the difference between residuals from a linear regression of calendar day on RMR as the dependent variable, there was no relationship between this variable and the number of days between the first and last measurements (*N*=27, β=−0.002, *t*=−0.50, *P*=0.62; Fig. 4B).

## DISCUSSION

The mean RMR that we measured was consistent with previous measurements of basal metabolic rates in *H. chrysoscelis* (Kamel et al., 1985; Lukose and Reinert, 1998), and was similar to that of other anuran species tested at similar temperatures after correcting for body mass (Wells, 2007). We found that RMR was repeatable in *H. chrysoscelis*, indicating that individual males consistently differ from one another in metabolic rate. Given that many advertisement call characteristics are also repeatable in this species (Reichert et al., 2024), we asked whether call repeatability is explained by variation in RMR. However, we found no evidence for a relationship between RMR and any call characteristics in male *H. chrysoscelis*, which indicates that the independent energy management model is the best explanation for the covariance between metabolic rate and calling in this species. Despite the consistent differences among individuals in RMR, RMR was highly variable, and we identified some factors that explain variation in this trait. These results have important implications for behavioral energetics in the context of sexual selection, which we discuss below.

### Effects of RMR on call characteristics

Variation in maintenance metabolic rates such as RMR is considered to be a potentially important driver of variation in behavior, but there are competing hypotheses in the form of different energy management models that make very different predictions for what the strength and direction of this relationship is expected to be (Careau and Garland, 2012; Mathot and Dingemanse, 2015). Empirical work to test these hypotheses has been similarly divided, with some work finding positive relationships between maintenance metabolism and behavioral expression, other studies finding negative relationships between these two traits, and some finding no relationship whatsoever, although a meta-analysis indicated the strongest support for positive relationships (Mathot et al., 2019). This raises the question of whether the relationship between RMR and behavior itself can be predicted somehow, perhaps based on the type of behavior involved. Mathot et al. (2019) found the strongest relationships between metabolic rates and behaviors for behaviors that are associated with energy gain or energy loss. Calling in anurans is highly energetically expensive and in many species is more energetically costly than any other measured behavior, and thus likely pushes animals close to their maximum metabolic rates (Prestwich, 1994; Wells, 2007). Because the energy management models are generally based on expected relationships between RMR and maximum metabolic rate, or aerobic scope (the difference between maximum and resting metabolic rates) (Auer et al., 2017), we had predicted that there would be a relationship between RMR and calling in *H. chrysoscelis*. One possible explanation for the discrepancy between this expectation and our results is that we measured a relatively short-term aspect of calling performance, the duration and rate of 30 consecutive advertisement calls, which usually amounted to a few minutes of calling. However, males can call for several hours on one night of chorusing and return to the chorus to call on several nights throughout the breeding season (Wells et al., 1995). These aspects of male calling represent longer-term energetic investment into signaling and may be more constrained by RMR. Although longer-term calling investment is much more challenging to measure, it is a worthy target of future study given the importance of chorus attendance for male mating success (Godwin and Roble, 1983; Ritke and Semlitsch, 1991; Sullivan and Hinshaw, 1992). More broadly, immediate short-term measurements of a display may better capture display power, rather than the overall energetic cost of displaying that is captured by nightly or seasonal calling output, and it is unclear which of these measures may be more likely to be related to RMR (Clark, 2012).

In a meta-analysis of relationships between maintenance metabolic rate and behavior, there was no evidence for covariation between courtship behaviors and metabolic rate, although the sample size of such studies was small (Mathot et al., 2019). For instance, in two studies of crickets, signaling effort and maintenance metabolic rate were not related (Ketola et al., 2009; Okada et al., 2011), although in another study with inbred crickets, some song traits were associated with resting metabolic rate (Ketola and Kotiaho, 2010). Selection may act to dissociate courtship behaviors such as advertisement calling from any limits imposed by maintenance metabolism because of their importance in determining reproductive success (Stoddard and Salazar, 2011). Because sexually selected signals are often highly energetically costly and in some cases reach the limits of performance, other aspects of metabolic rate more directly linked to performance such as the maximum metabolic rate or metabolic efficiency may be more important determinants of the expression of sexually selected traits (Hill, 2011; Koch and Hill, 2018; Koch et al., 2021; Podos, 2022; Watson and Lighton, 1994).

Lack of a relationship between calling behaviors and RMR has important implications for the understanding of sexual selection in *H. chrysoscelis*. In particular, many models of sexual selection argue that females prefer male traits indicating their overall quality or condition (Cotton et al., 2004; Rowe and Houle, 1996). To the extent that RMR is associated with condition or quality, this means that female preferences for traits such as call duration are unlikely to function to allow females to select males with certain RMR phenotypes. This is especially surprising given that RMR was repeatable, and thus potentially heritable and a target of indirect female preferences for male genetic quality (Kirkpatrick and Ryan, 1991). However, it is unknown whether RMR is related to condition, survival or other aspects of male quality in *H. chrysoscelis*. Although maintenance metabolic rates often covary with fitness (Larivée et al., 2010), they may be less likely to do so in species such as *H. chrysoscelis* because their maintenance energy requirements are very low, they are relatively inactive and they use anaerobic substrates for locomotion. As above, it may also be that female preference is targeting other aspects of male metabolic phenotypes that are more directly related to calling, which is an important target for future studies.

Although a meta-analysis found no significant effects of whether test subjects were wild caught versus lab reared on the relationship between behavior and metabolic rate (Mathot et al., 2019), other factors related to using a wild population may have affected our estimates of either RMR or calling. One caveat to our findings is that because we recorded male calling in the field, we could not control for social context. When faced with social competition, male *H. chrysoscelis* increase call duration and decrease call rate (Reichert et al., 2024; Ward et al., 2013), and in *H. versicolor*, extreme levels of competition result in males increasing call effort, which should increase energetic expenditure (Reichert and Gerhardt, 2012; Wells and Taigen, 1986). However, a previous study found that there are still consistent among-individual differences in *H. chrysoscelis* call characteristics even for recordings made in the field under varying social environments (Reichert et al., 2024). Nevertheless, variation in social context could have introduced variation in our measurements of call characteristics that obscured any relationship with RMR. Similarly, because we recorded spontaneous calling, males were not necessarily calling at their upper performance limit, where a relationship with RMR may be more likely.

### Factors affecting variation in RMR

Although RMR does not appear to have influenced calling behavior, RMR decreased linearly over the course of the breeding season in both 2023 and 2024. Interestingly, both this study and previous work (Reichert et al., 2024) found that some call characteristics also varied over the course of the breeding season in *H. chrysoscelis*, and previous work in *H. versicolor* found similar patterns (Runkle et al., 1994). The decrease in RMR as the breeding season progressed could be explained by several variables that co-vary with time of year, and experimental manipulations would be needed to determine the specific mechanism, but such a seasonal change in metabolic rate is common across taxa (McClune et al., 2015; McKechnie and Swanson, 2010; Podhajský and Gvoždík, 2016). First, photoperiod increases over the majority of the breeding season of *H. chrysoscelis*, and there is some evidence in other species for an effect of photoperiod on maintenance metabolic rates (Hutchison and Kohl, 1971; Lanciani et al., 1990; Warner et al., 2010). Second, temperature also increases over the course of the breeding season, and this can result in acclimation that can affect metabolic rates (Dubois et al., 2016). Third, metabolic rates are affected by reproductive state, and although males call throughout the breeding season, there are likely changes in hormone levels, energy stores and nutritional status over this time period (Gingerich et al., 2010; O'Bryant and Wilczynski, 2010; Sparling et al., 2006; Wells and Bevier, 1997), which correspond to changes in calling activity as the season progresses (Runkle et al., 1994).

Some aspects of the experimental design may have influenced RMR measurements. First, our acclimation period prior to recording was 15 min, followed by a 15 min recording from the baseline chamber. The first RMR measurement was therefore recorded only 30 min after the frog was placed in the chamber, which may have been too short for any effects of handling on metabolic rate to have dissipated. There is mixed evidence for such handling effects in other frog species (Cabanac and Cabanac, 2000; Narayan et al., 2012). Nevertheless, because we performed three repetitions for each individual and used the lowest mean *V̇*_O_2__ as our estimate of RMR, this should reduce any variability introduced by handling or other conditions of the experiment. There was no evidence that *V̇*_O_2__ decreased across the three repetitions and in fact *V̇*_O_2_ _was lowest in the first repetition in 57% of our recordings. Thus, although we cannot completely rule out effects of handling, they appear to be minimal in our dataset. Second, we cannot guarantee that individuals were post-absorptive because individuals were always in captivity less than 24 h before their RMR was measured. Although digestion may have increased the measured metabolic rate and introduced some variation among individuals, the metabolic rate we measured is the relevant measurement for the question we are trying to address: whether baseline levels of metabolism expressed during the breeding season are correlated with sexual signaling behavior.

Despite seasonal and other sources of variation in RMR, we found that RMR was significantly repeatable, with a repeatability coefficient (0.4) that is consistent with the typical range of repeatabilities of metabolic rates across taxa, including those measured in free-living animals (Auer et al., 2016; Nespolo and Franco, 2007). This indicates that even in this ectothermic species with relatively low maintenance energy requirements, individuals vary substantially in the amount of energy devoted to maintenance even when controlling for variation in body mass. In addition, although we found no effects of RMR on calling, individual variation in RMR could still have consequences for the amount of time needed to spend on foraging versus other activities, and could also lead to variation in growth, condition or other aspects of behavior (Biro and Stamps, 2010). Repeatability of RMR also indicates the potential for this trait to be heritable and respond to selection (Pettersen et al., 2018). It is unknown whether there is selection on maintenance metabolism in *H. chrysoscelis*, as there is in other taxa (Artacho et al., 2015; Boratyński and Koteja, 2010; Nilsson and Nilsson, 2016). We also investigated whether repeatability declines with time between measurements, a common observation for measurements of both behavior and metabolic rates (Bell et al., 2009; White et al., 2013). Although our haphazard sampling of field-caught individuals meant that we could not directly address whether repeatability itself is affected, we did find that RMR measurements of the same individual were more variable with greater time between the measurements. However, this effect was largely because RMR itself is not consistent over the breeding season; when we accounted for the decrease in RMR with calendar day then there was no longer any indication that RMR measurements became less consistent with more time between measurements. It remains an open question whether individuals differ in the plasticity of their RMR over time (Dingemanse et al., 2010), and whether RMR is still repeatable with even longer time intervals between measurements (e.g. across years).

### Conclusions

Metabolism undoubtedly affects calling at some level in anurans, but there are major open questions about what aspects of metabolic processes determine variation in calling and whether metabolism can explain differences among individuals or among species in call characteristics. Our findings indicate that maintenance metabolism is not a major driver of repeatable variation in energetically costly call characteristics, and we can speculate that variation in calls may be more strongly related to maximum metabolic rates, which should be investigated in future studies. Nevertheless, our findings of repeatable variation in RMR indicate that this trait also varies among individuals, which could have consequences for other traits more closely tied to RMR and for any selection that may be acting on RMR itself. Given the close connection between energy use and behavior, future work that focuses on multiple aspects of these traits has a strong potential to reveal the mechanistic drivers of variation in behavioral phenotypes.

## Supplementary Material

10.1242/jexbio.250570_sup1 Supplementary information
