# Supplementary material for: Resting metabolic rate is repeatable, but does not affect call characteristics, in the gray treefrog Hyla chrysoscelis
Source: J Exp Biol. 2025 Jul 23;228(15):jeb250570. doi: 10.1242/jeb.250570 (PMC12319408; doi:10.1242/jeb.250570)
Supplement: Supplementary information [file jexbio-228-250570-s1.pdf]

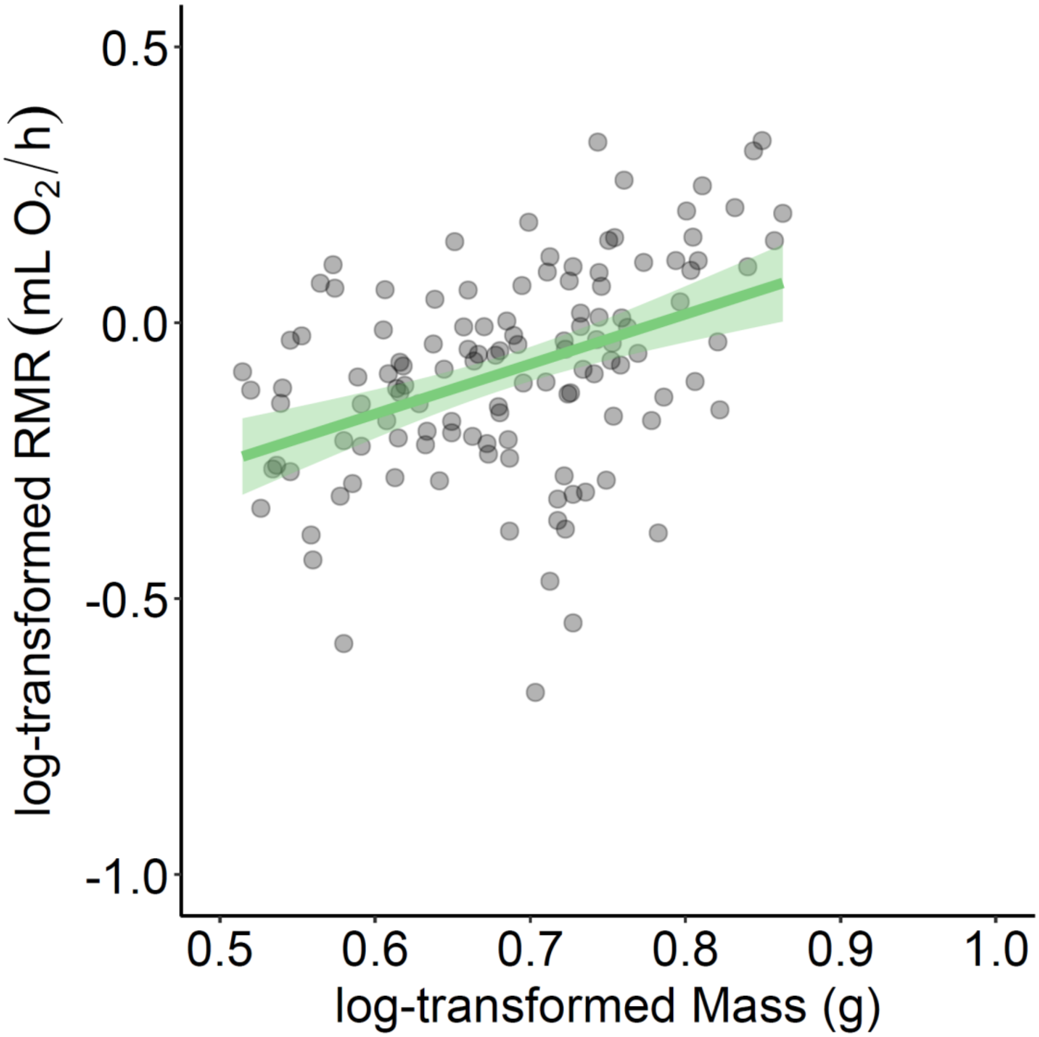

Fig. S1. The relationship between body mass and RMR for male *H. chrysoscelis* when both mass and RMR were log_{10}-transformed. Each point represents an individual's mass at the time that their RMR was measured and the trend line shows a positive, linear relationship. The shaded area around the line represents the 95% confidence interval. Note that some individuals contributed multiple data points because they were captured and measured more than once, so the regression line is for illustration purposes only. The relationship between the variables is not obviously improved by the log-transformation compared to the untransformed data (compare with Fig. 2B in main text).

Journal of Experimental Biology • Supplementary information

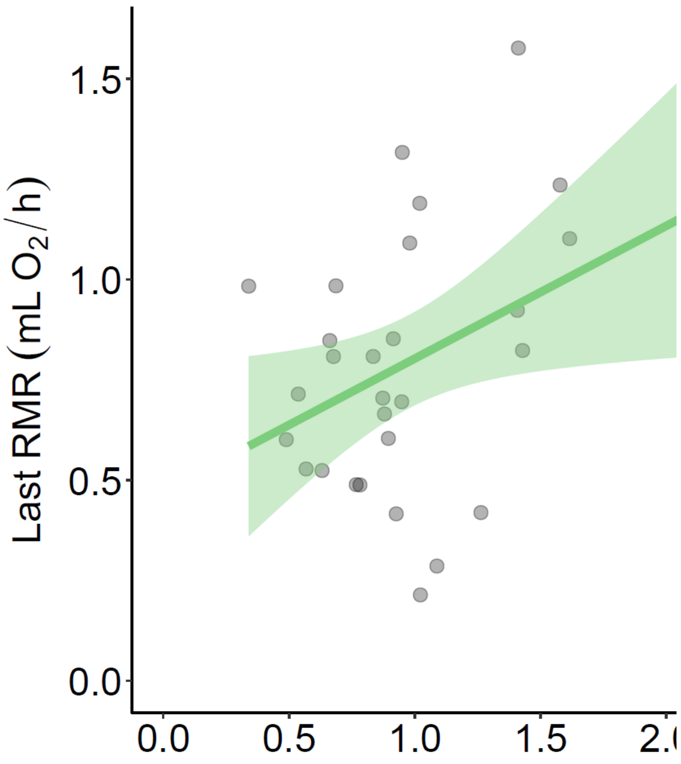

Fig. S2. Illustration of consistent among-individual differences in RMR as a scatterplot. Each point represents an individual and corresponds to the first and last measurement of its RMR. Trend line is from a linear least squares regression to illustrate the pattern; the shaded area around the line represents the 95% confidence interval. *N* = 29 individuals. This plot shows raw values of RMR. Compare to Fig. 3 in the main text, which illustrates RMR as the residuals of RMR after accounting for the fixed effects of mass and calendar day from a mixed model with individual identity as a random effect.

Journal of Experimental Biology • Supplementary information

Table S1. Repeatability of call traits.

Characteristic	R	s.e.m.	*P*
Call duration	0.287	0.167	0.015
Pulse number	0.244	0.162	0.047
Call rate	0.254	0.166	0.0384
Call effort	0.191	0.159	0.103
Dominant frequency	0.606	0.130	0.006

The repeatability coefficient (R) was calculated from a linear mixed model using the rptR 0.9.22 package with the mean value of the call characteristic for a given recording as the dependent variable (Gaussian), body temperature as a factor, and individual identity as a random effect.

Table S2. Inter-observer reliability of measurements of call traits.

Characteristic	r	β	s.e.m.
Call duration	0.975	0.973	0.0025
Pulse number	0.957	0.952	0.0055
Call rate	0.953	0.955	0.0059
Call effort	0.915	0.898	0.0077
Dominant frequency	0.995	0.996	0.0022

The correlation coefficient r is given along with the slope ($\beta \pm$ s.e.m.) for a least squares regression of the call characteristic measured for each call compared between the two observers, for 92 recordings from 2023.

Journal of Experimental Biology • Supplementary information
